# Development and Evaluation of Image Reconstruction Algorithms for a Novel Desktop SPECT System

**DOI:** 10.22038/aojnmb.2017.8708

**Published:** 2017

**Authors:** Navid Zeraatkar, Arman Rahmim, Saeed Sarkar, Mohammad Reza Ay

**Affiliations:** 1 Research Center for Molecular and Cellular Imaging, Tehran University of Medical Sciences, Tehran, Iran; 2 Department of Radiology, Johns Hopkins University, Baltimore, Maryland, US; 3 Department of Electrical and Computer Engineering, Johns Hopkins University, Baltimore, Maryland, US; 4 Department of Medical Physics and Biomedical Engineering, Tehran University of Medical Sciences, Tehran, Iran; 5 Research Center for Science and Technology in Medicine, Tehran University of Medical Sciences, Tehran, Iran

**Keywords:** ART, Image reconstruction, MLEM, OSEM, PERSPECT

## Abstract

**Objective (s):**

Various iterative reconstruction algorithms in nuclear medicine have been introduced in the last three decades. For each new imaging system, it is wise to select appropriate image reconstruction algorithms and evaluate their performance. In this study, three approaches of image reconstruction were developed for a novel desktop open-gantry SPECT system, PERSPECT, to assess their performance in terms of the quality of the resultant reconstructed images.

**Methods:**

In the present work, a proposed image reconstruction algorithm for the PERSPECT, referred to as *quasi-simultaneous multiplicative algebraic reconstruction technique* (qSMART), together with two popular image reconstruction methods, *maximum-likelihood expectation-maximization* (MLEM) and *ordered-subsets* EM (OSEM), were implemented and compared. Analytic and Monte Carlo simulations were applied for data acquisition of various phantoms including a micro-Derenzo phantom. All acquired data were reconstructed by the three algorithms using different number of iterations (1-40 ). A thorough set of figures-of-merit was utilized to quantitatively compare the generated images.

**Results:**

OSEM depicted reconstructed images of higher (or matching) quality in comparison to qSMART. MLEM also reached nearly similar quality as OSEM but at higher number of iterations. The graph of data discrepancy revealed that the ranking of the three approaches in terms of convergence speed is as qSMART, OSEM, and MLEM. Furthermore, bias-versus-noise curves indicated that optimal bias-noise results were achieved using OSEM.

**Conclusion:**

The results showed that although qSMART can be applied for image reconstruction if being halted in the early iterations (up to 5), the best achievable quality of images is obtained using the OSEM.

## Introduction

Given drawbacks of analytic image reconstruction methods, dominantly *filtered back projection* (FBP), iterative reconstruction methods have been becoming the prominent methods for image reconstruction in SPECT and PET (1). Analytic image reconstruction methods commonly utilize simplistic models of emission and detection processes, and the resulting images suffer from streak artefacts and noise. By contrast, iterative reconstruction techniques can incorporate sophisticated models for emission and detection, including statistical properties of the emission process, attenuation, scatter, detector response function, etc. The various iterative image reconstruction approaches can be divided into two main categories: those considering the statistical nature of the data and the ones that do not (2). One of the most common algorithms in the first category is *maximum-likelihood expectation-maximization* (MLEM) (3, 4) in which Poisson distribution is considered for emission process. In MLEM, the superposition of the differences between all the projection rays and the forward projection of the estimated image are used for updating the image. Since a main challenge in iterative reconstruction is the convergence speed, the accelerated version of MLEM which is known as *ordered-subsets expectation-maximization* (OSEM) (5) is also a popular option in nuclear medicine image reconstruction. In each subiteration of OSEM, a subset of projections are used for updating leading to faster convergence in comparison to MLEM; a full iteration is completed when all subsets are used. *Algebraic reconstruction technique* (ART) (6) belongs to the second category of non-statistical iterative reconstruction methods. ART is identified as a row-action method where the difference between the estimated image and the measured projection for each projection ray is used for updating the image voxels on the path of that ray (2). Given the numerous updates (equal to the number of views) in each reconstruction iteration, ART can be a fast algorithm. However, it is susceptible to noise in particular in the case of multiplicative ART (MART) in which the update is performed by multiplication of the error. It should be remarked that the simultaneous version of MART (SMART) is similar to the MLEM method (2). More details about the abovementioned reconstruction approaches are provided in *Methods*.

PERSPECT is a desktop open-gantry system whose design concept was recently proposed (7, 8). It consists of an imaging desk together with a tilted collimator-detector pair (the head) which is located beneath the desk. The head rotates around the object in a step-and-shoot manner for data acquisition. For image reconstruction, an algorithm entitled *Finite-Aperture-Based Circular Projections* (FABCP) was developed and applied through a forward/backward projector pair in a subsetized MLEM (subset size of one) algorithm which can be also considered as an MART-based method.

In this study, we developed two other image reconstruction algorithms to compare their performance in terms of image quality as applied to the PERSPECT relative to the original proposed image reconstruction algorithm. Both Monte Carlo (MC) and analytical simulations were utilized for data acquisition. The acquired data of various phantoms were then reconstructed using the three reconstruction codes. In addition to visual comparisons of the reconstructed images, some quantitative figures of merit were calculated. Furthermore, image reconstruction was performed at different number of full iterations to assess the effect of number of iterations on the resultant image quality.

## Methods

### Data Acquisition

**Figure 1:**
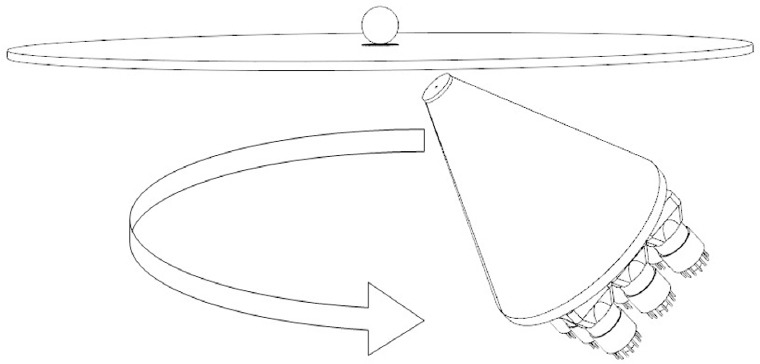
A schematic view of the PERSPECT with pinhole collimator. The arrow shows the rotation path of the head beneath the imaging desk

PERSPECT is a newly patented concept for desktop SPECT imaging of small animals or small organs of human. In contrast to the conventional SPECT systems with a rotating gantry which usually leads in a closed-gantry structure, the PERSPECT provides an open imaging desk for locating the object to be imaged. A head rotates around the object underneath the desk with a tilt angle in a step-and-shoot manner for data acquisition in different views (7, 8). Although the system is capable of applying any type of collimator and/or detector, pinhole collimator together with scintillator crystal detector have been already assessed and showed to have acceptable results (8). Figure 1 represents a schematic view of the PERSPECT scanner.

For the majority of the measurements in this work, MC simulations were used for data acquisition. *Geant4 Application for Tomographic Emission* (GATE) toolkit (version 6.1) (9, 10) was applied for this aim. For all MC simulations, we applied a double knife-edge pinhole collimator with diameter of 1 mm, opening angle of 56°, and thickness of 5 mm. A sodium iodide thallium-activated [NaI(Tl)] crystal with the size of 30 cm×30 cm×3/8 in. was considered as the scintillator detector. Energy resolution, intrinsic spatial resolution, and energy window were respectively simulated as 10% at 140 keV, 3 mm, and 125-155 keV. Likewise, detector tilt angle, pinhole-to-center-of-image-matrix distance, and pinhole-to-detector distance were set to 30°, 18.75 mm and 30 cm, respectively. Projection data were stored in 512×512 matrices. For all assessments reported in this work, the abovementioned parameter values were utilized for the data acquisition, except if stated otherwise.

For spatial resolution calculation, a point source with activity of 740 kBq was simulated at the center of field-of-view (FOV). Furthermore, to compute normalized squared error (NSE), as a measure of similarity between the reconstructed image and the hypothetical reference image, noise [in terms of percentage standard deviation (STD%)], non-uniformity, and bias, a spherical phantom with diameter of 10 mm including a total activity of 103.6 MBq (Tc-99m) with uniform concentration was simulated at the center of the FOV. Moreover, to measure contrast and contrast-to-noise ratio (CNR), two concentric spheres with diameters of 10 mm and 3 mm were used as the background and the hot regions, respectively. Both spheres had uniform activity concentration of Tc-99m with values leading to hot-to-background activity concentration of 4.7.

Furthermore, a micro-Derenzo phantom was simulated using the analytical simulator introduced in Ref. (8). The phantom was defined as a voxelized source phantom in a 201×201×201 matrix with voxel size of (0.1 mm)^3^. It consists of a cylinder 20 mm in both diameter and thickness with six sections each containing some hot rods with diameters of 1.2 mm, 1.7 mm, 2.5 mm, 3.3 mm, 4.2 mm, and 4.9 mm. The center-to-center distance of the rods in each section is twice the corresponding rods’ diameter. Total activity of the rods was considered as 74 MBq. For scanning, the phantom was simulated as rods were perpendicular to the imaging desk. Also, some scan parameters were changed from what was set for MC simulations. The different parameters were pinhole diameter, tilt angle, and detector size that were respectively set to 0.5 mm, 40°, and 50 cm×50 cm.

For all the above scans, data acquisition was performed via 16 views over a 360° angular span with scan time of 60 s per view (120 s per view for the micro-Derenzo phantom).

It is remarked that since the attenuation/scatter effects are not significant (nor of interest) for small-size objects like the ones used in the current study, no attenuating/scattering medium was simulated in the scans to gain ease and short simulation time.

### Image Reconstruction Approaches

Three different image reconstruction algorithms were implemented in a C++ platform to assess image quality performance. The FABCP algorithm (8) was applied for developing the forward-backward projector pair in all three image reconstruction methods.

FABCP method for pinhole systems is based on modeling the pinhole by a finite aperture with a *resolution-related effective diameter* (*d_re_*) which is calculated as (11, 12):


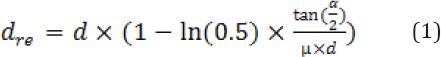


where *d*, *µ*, and *α* denote the physical diameter of the pinhole, linear attenuation coefficient of the collimator, and the opening angle of the pinhole, respectively. In the current work, geometric efficiency of the collimator was also modeled in the image reconstruction algorithm. For implementing this, the geometric efficiency (*E*) of the collimator corresponding to each image voxel was calculated and taken into account through the forward projection step using Equation 2 (13):


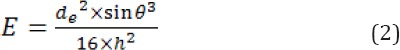


where *θ* is the incidence angle of the hypothetical line connecting the voxel to the center of the aperture on the aperture plane, *h* is the distance of the voxel from the pinhole, and *d_e_* denotes the *sensitivity-related effective diameter* of the pinhole formulated as (11, 14-18):


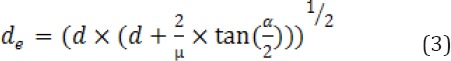


From the algorithm classification point of view in iterative image reconstruction, the image reconstruction algorithm introduced in Ref. (8) (as a subsetized MLEM) can also be assumed as a quasi-SMART method where the expected projections are first computed by forward projection of the current estimate of the image (an initial estimate at the first view of the first iteration). The ratio of the measured projections to the expected projections then is calculated and backprojected to the current estimated image to update it and generate the new estimate. The forward and backward projection steps are performed using the projector-backprojector pair. In ART, the process is performed in a ray-by-ray manner and all image voxels along the ray are updated. However, in the original image reconstruction of the PERSPECT, all projection bins corresponding to each image voxel are considered for update while the process is performed view-by-view; this is why we call it quasi-SMART (qSMART). The general MART algorithm is formulated as (2):


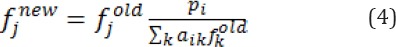


where 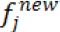 and 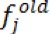 refer to the current and previous estimates of the *j*^th^ voxel in the image, respectively. In addition, *a_ik_* and *p_i_* denote the elements of system matrix and projection matrix, respectively.

The qSMART algorithm can be formulated as:


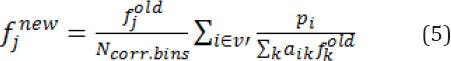


Here, *v’* denotes the projection bins of view *v* corresponding to image voxel *f_j_* (based on the FABCP algorithm) and *N_corr. bins_* are the number of bins in *v’*.

Moreover, MLEM and OSEM approaches were developed using the FABCP forward-backward projector pair. The MLEM method is formulated for a single voxel as (2-4):


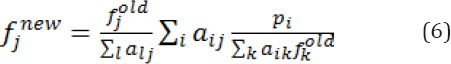


In a similar way, the OSEM can be formulated by (2):


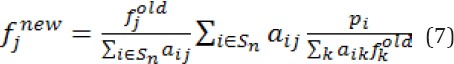


here, *S_n_* denotes the subsets and *n∈N* is the subset size.

It is remarked that Eq. 6 and 7 provided above are the general formulas of respectively MLEM and OSEM. However, in the current work applying FABCP algorithm through forward/backward projection pair, they alter slightly when implementing.

Noticing that using four subsets with the least correlation can lead to faster convergence (2, 19), we used the projection view data in an order having four equally-spaced subsets through angular span of the scan i.e. four subsets with subset size of four for the current data acquisition setup.

It is worth noting that the developed OSEM with subset size of one is similar to qSMART.

All the datasets were reconstructed with all the three reconstruction approaches including qSMART, MLEM, and OSEM using image matrix size of 101×101×101 and voxel size of (0.2 mm)^3^. The initial estimate was a 3D all-ones matrix. To assess the image quality against the number of iterations, the number of full iterations was changed from 1 to 40 and all figures of merit of image quality (see *next subsection*) were computed for each iteration number.

### Figures of Merit

To calculate spatial resolution along each direction (*x*, *y*, and *z*), all slices of the reconstructed images along the other two directions were summed to form the count profile along the specified direction. A Gaussian was fitted on each count profile, and its full-width at half-maximum (FWHM) was considered as the resolution along the given direction. FWHM values along the three directions then were averaged to form the mean spatial resolution.

To compute non-uniformity and STD%, a sphere 3/4 in diameter as the considered uniform-activity sphere was drawn on the reconstructed image as the volume-of-interest. Non-uniformity (in percentage) was calculated as:


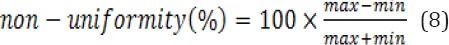


where *max* and *min* denote the maximum and the minimum values in the volume of interest (VOI), respectively.

Moreover, bias, as a measure of systematic error, was computed in the VOI introduced above as:


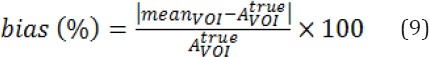


Here, *mean_VOI_* and 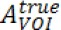 stand for mean value of the VOI and total activity of the VOI in the phantom, respectively.

Using bias and STD% (as a measure of noise) at different number of iterations, bias-noise curves were plotted for each reconstruction method to provide an important scale of the performance of the corresponding reconstruction algorithm.

Furthermore, contrast and CNR were measured in the corresponding reconstructed images as:


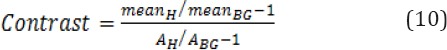



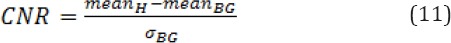


in which *mean_H_*, *mean_BG_, A_H_/A_BG_*, and *^α^BG* are the mean values of the hot and the background regions, the actual concentration ratio between the hot and the background regions, and the standard deviation of the background region, respectively.

NSE for each resultant image was calculated as:


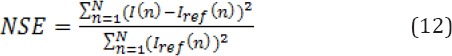


Here, *I*, *I_ref_*, *n*, and *N* are the reconstructed image matrix, the reference image matrix, the voxel index number, and the total number of voxels in the matrix, respectively. The reference matrix was a 3D image matrix with the size of the reconstructed image matrix containing a 10 mm-diameter sphere. Prior to the calculation, both the reconstructed matrix and the reference matrix were normalized to the mean value of their own 3D matrix.

The reconstructed images of the micro-Derenzo phantom were used for obtaining *data discrepancy* between the forward projection of the reconstructed image and the measured projections as a measure of convergence. Data discrepancy was computed as mean squared error (MSE) in the projection-space:


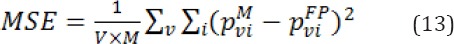


where *V* and *M* are respectively total number of views and projection matrix size, and 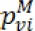 and 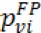 denote *i^th^* bin at *v^th^* view in the measured and forward-projected projection matrix, respectively.

## Results

**Figure 2:**
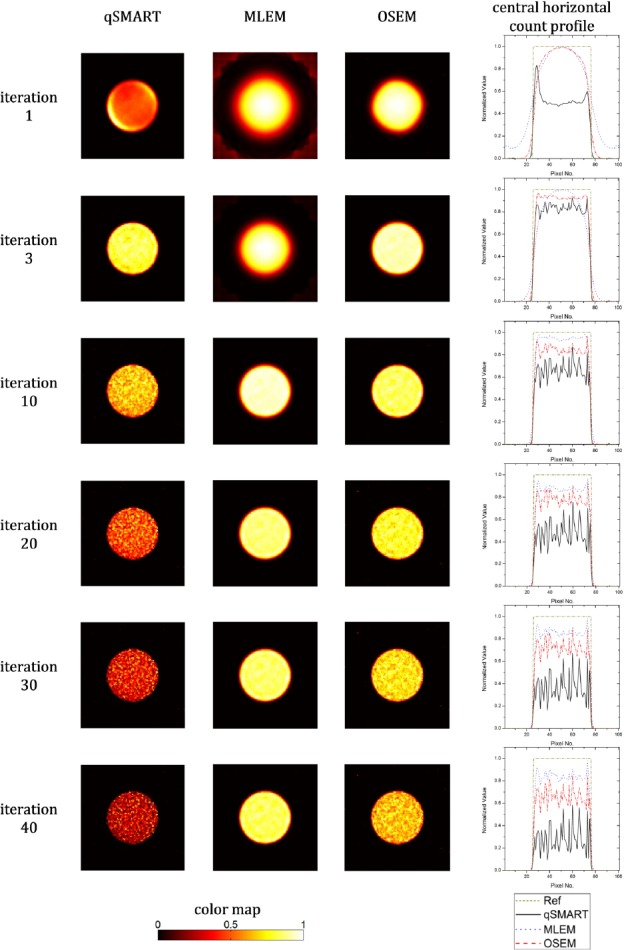
The central slice of the reconstructed images of the 10 mm-diameter sphere reconstructed by qSMART, MLEM, and OSEM methods after 1, 3, 10, 20, 30, and 40 full iterations. The images are normalized to the maximum value of the corresponding slice. The central horizontal count profiles of the images together with the one of the corresponding hypothetical reference image are also plotted

Figure 2 shows the central slice of the recon-structed images of the 10 mm-diameter sphere filled with uniform activity concentration reconstructed by the three reconstruction approaches introduced earlier at different numbers of full iterations. Also, the central horizontal count profiles of the shown images together with the one of the hypothetical reference image were plotted. It can be seen that MLEM and OSEM can reach better images. While the best image for OSEM occurs at third iteration, it happens later for MLEM after 10 iterations.

**Figure 3:**
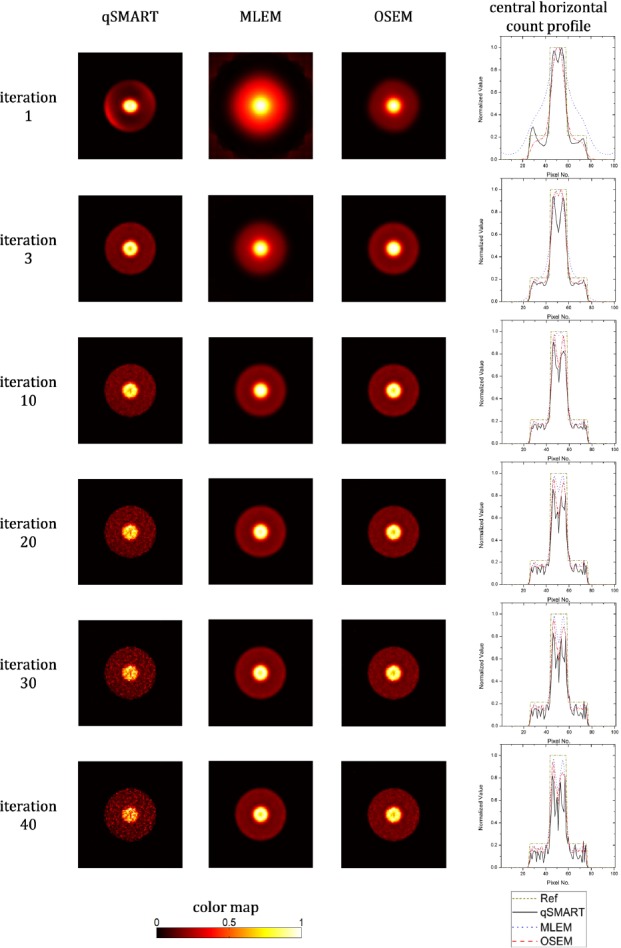
The central slice of the reconstructed images of the contrast phantom reconstructed by qSMART, MLEM, and OSEM methods after 1, 3, 10, 20, 30, and 40 full iterations. The images are normalized to the maximum value of the corresponding slice. The central horizontal count profiles of the images together with the one of the corresponding hypothetical reference image are also plotted

Similarly, the central slice of the reconstructed images of the phantom used for measurement of contrast and CNR (a hot sphere inside a background sphere) reconstructed by the three reconstruction methods at different numbers of full iterations are shown in Figure 3. The central horizontal count profiles of each image are also sketched in addition to the corresponding count profile of the hypothetical reference image. The images of qSMART are deteriorated by noise effects with increasing the number of iterations preventing it from reconstructing good images in comparison to MLEM and OSEM. OSEM reconstructed the best image after three full iterations. MLEM reached its best results after 10 iterations.

**Figure 4:**
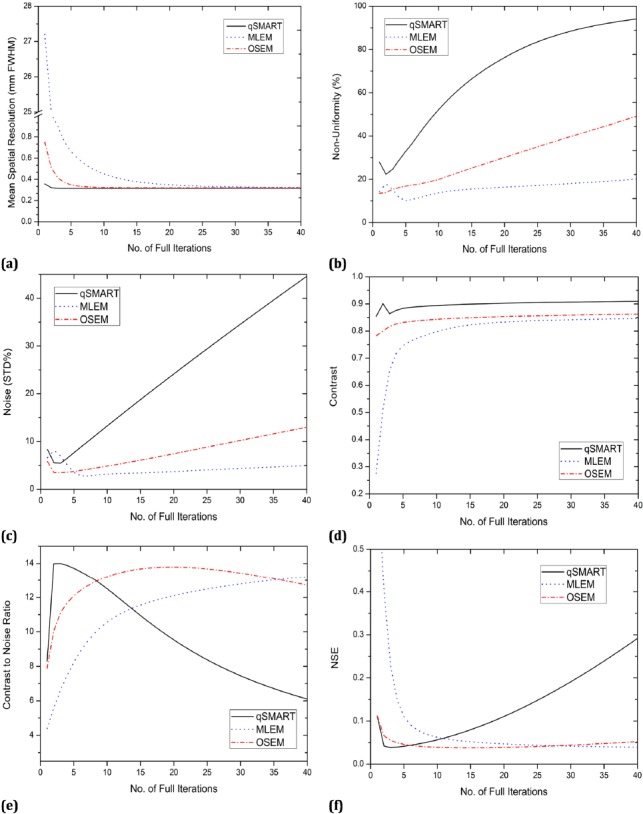
Spatial resolution, non-uniformity, noise, contrast, CNR, and NSE calculated for the reconstructed images by qSMART, MLEM, and OSEM against the number of full iterations

Figure 4 demonstrates spatial resolution, non-uniformity (percentage), noise (in terms of STD%), contrast, CNR, and NSE for the three image reconstruction algorithms. All parameters were computed and plotted against the number of full iterations. As a general observation, the trend of graphs relating to MLEM at higher number of iterations is similar (not the same) to the corresponding ones of OSEM at lower number of iterations that would be expected noting that OSEM is the accelerated version of MLEM.

**Figure 5:**
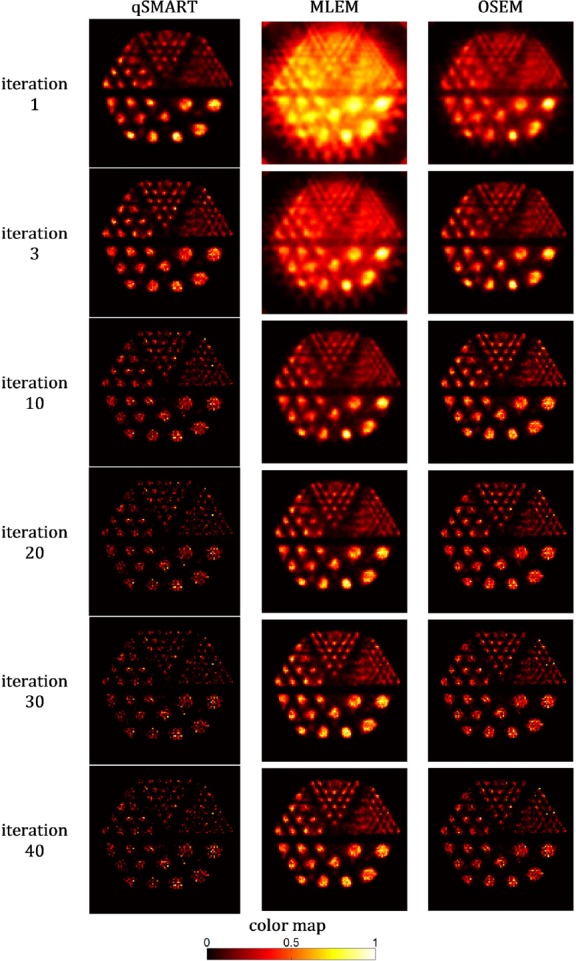
The central slice of the reconstructed images of the micro-Derenzo phantom reconstructed by qSMART, MLEM, and OSEM methods after 1, 3, 10, 20, 30, and 40 full iterations. The images are normalized to the maximum value of the corresponding slice

To provide a qualitative performance assessment of the different image reconstruction algorithms on the resultant images at various numbers of iterations using a rather complicated phantom, the reconstructed images of the micro-Derenzo phantom are shown in Figure 5. The images correspond to the central slice of the image matrix and are normalized to the maximum value of the slice. The best results can be seen for OSEM after three iterations and MLEM after 20 iterations resolving even the smallest section of the phantom (1.2 mm) with appropriate quality.

**Figure 6:**
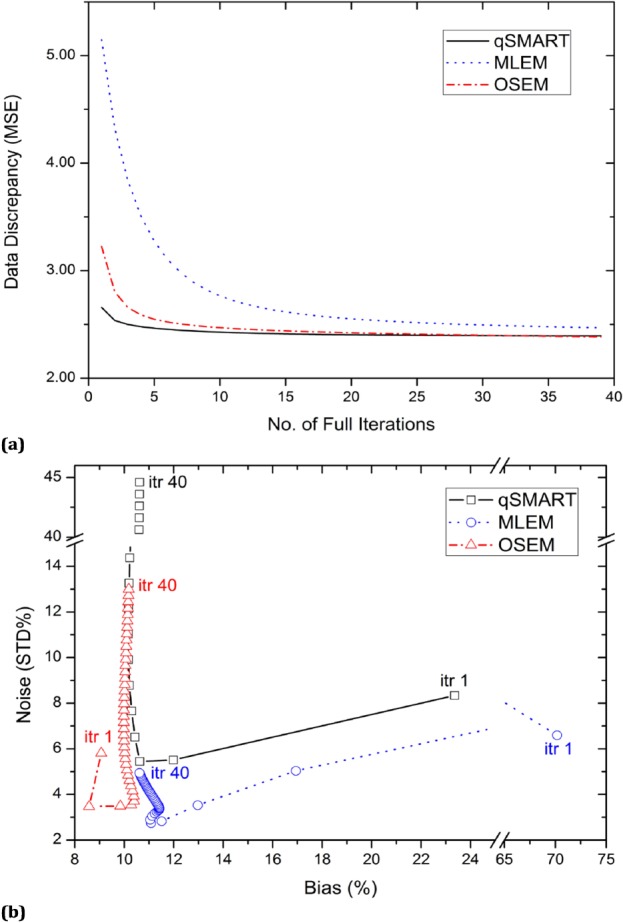
Data discrepancy (in terms of MSE) for qSMART, MLEM, and OSEM against the number of full iterations (a) and bias-noise curves for qSMART, MLEM, and OSEM (b). For bias-noise curves, the point corresponding to the obtained result of every iteration is indicated by a symbol (*square* for qSMART, *circle* for MLEM, and *triangle* for OSEM) on the curves. Also, the first iteration (*itr* 1) and the last assessed iteration (*itr* 40) are shown for each curve

The projection data and the reconstructed images of the micro-Derenzo phantom were also used for data discrepancy calculations. Figure 6.a shows the discrepancy (calculated as MSE) between the measured data and forward-projection-induced data against the number of iterations for qSMART, MLEM, and OSEM, respectively. The flatness of the graph can be translated to convergence. However, the scale of the variations (in vertical axis) should be taken into account for this regard.

The bias-noise curves of the three reconstruction approaches are plotted in Figure 6.b. As shown, OSEM and MLEM can yield better bias-noise pair values in comparison to qSMART.

## Discussion

The primary goal of this study was to evaluate the performance of two newly-developed image reconstruction methods, MLEM and OSEM (with 4 subsets), versus the previously introduced algorithm, qSMART, for a desktop open-gantry SPECT system, the PERSPECT. All the mentioned reconstruction approaches were implemented as some in-house codes. MLEM and OSEM algorithms were applied as two classical methods through a dedicated implementation for the case of the PERSPECT. FABCP algorithm was applied for development of forward/backward projector pair in all the three reconstruction methods. MC simulation was used for the vast majority of the data acquisition scans. However, a pre-verified analytical model (8) of the system was used for simulation of the micro-Derenzo phantom. The acquired projection data corresponding to each phantom were reconstructed by the three reconstruction codes independently, using different number of full iteration numbers up to 40. In addition to provide the sample slices of the reconstructed images as a sense of quality, a thorough set of quantitative parameters were calculated for each reconstruction method at each iteration number. In the following, the obtained results reported in the previous section are discussed.

In general, ART has a relatively low computational effort in comparison to statistical-based iterative reconstruction techniques (2). This is mainly due to numerous update processes performed through each iteration of ART. However, the multiplicative approach of updating in MART introduces the disadvantage of noise susceptibility for it. With respect to the noisy data in nuclear medicine, such a susceptibility to noise is translated into noisier resultant images with increasing the number of iterations. It should be stated that such a noise amplification characteristics against the number of iterations is also seen in MLEM and its accelerated version, OSEM, regarding the multiplicative approach of updating applied. However, noting that in MART the image is updated by every projection ray in each iteration while in MLEM (and OSEM) such updates are performed once in every iteration (subiteration), noise handling performance of MLEM and OSEM is expected to be better than MART methods.

As seen in Figure 2 from the visual point of view, whereas OSEM produces a good image after three iterations, MLEM still needs more iterations to reach a satisfactory result demonstrating the expected acceleration of OSEM over MLEM. MLEM finally reconstructed its best result after ten iterations. The reconstructed image of OSEM at the third iteration is almost similar to the one of MLEM at the tenth iteration regarding the count profiles. Noise amplification harshly affects the image quality in qSMART with increasing the number of iterations causing qSMART to reconstruct its best image after three iterations while it is still poorer than the best ones of OSEM and MLEM. OSEM images also gets noisier at higher number of iterations. MLEM, regarding its fewer updates in each iteration, amplifies noise at high iteration numbers less than the other methods but showing a very slow convergence speed.

Similar to the trend seen in Figure 2, the reconstructed images in Figure 3 for the contrast phantom show that OSEM achieves the best answer after three iterations. qSMART also reaches its best result after three iterations which is poorer than the one of OSEM. MLEM, demonstrating its relative slowness, converges to an acceptable image after ten iterations. Noise enhancement of the all reconstruction approaches versus increasing the iteration number can be clearly seen while MLEM and qSMART has respectively the least and the most susceptibility to noise.

Figure 4 illustrates most of the calculated figures of merit of the images reconstructed by qSMART, MLEM, and OSEM versus the number of iterations. Figure 4.a shows that qSMART reaches its final achievable spatial resolution with very low computational effort: only two iterations. OSEM reaches the same spatial resolution as what qSMART achieved after nine full iterations. In contrast, MLEM reconstructed such results after about 35 iterations while its resultant spatial resolution at iterations below five is very poor. Regardless of the required number of iterations, all methods can accomplish the same spatial resolution.

Figure 4.b reveals that the uniformity of the reconstructed images by OSEM monotonically decreases (i.e. non-uniformity increases) with iteration number. Such a trend is seen similarly for MLEM and qSMART after respectively the fifth and the second iteration. The best achieved uniformity value (non-uniformity of 10.1%) belongs to MLEM method at the fifth iteration. While qSMART reconstructed more non-uniform images with higher slope non-uniformity graph against number of iterations, the best images in terms of uniformity, also having the most deliberate ascending non-uniformity correspond to MLEM. It should also be noted that in the first three iterations, the uniformity of the images reconstructed by OSEM are better than the ones of MLEM.

Figure 4.c shows that the minimum amount of noise (STD% of 2.7%) is achieved using MLEM at the seventh iteration. The best result of qSMART and OSEM in terms of noise happens at the third and the second iteration, respectively. After experiencing the minimum point, the noise level of the resultant images of all methods increases with increasing the number of iterations. However, MLEM has the least susceptibility to noise whereas qSMART has the worst behavior against noise. The fact that MLEM (and its accelerated version, OSEM) introduces noise to the reconstructed images in particular at higher number of iterations is well known in the literature (2, 20, 21). As explained earlier, regarding the numerous updates within every full iteration in qSMART and the multiplicative nature of the update, the deterioration of the reconstructed images by noise with increasing the number of iterations is more sever in qSMART.

The contrast of the reconstructed image of the corresponding phantom using the three reconstruction methods versus iteration number is depicted in Figure 4.d. As shown, the highest contrast is obtained by qSMART with approximate value of 0.9, 10% less than the highest achievable value. OSEM reaches its highest contrast value (0.86) at 23^rd^ iteration. MLEM continues reconstructing images of higher contrast with increasing the number of iterations reaching the value of 0.85 at the 40^th^ iteration. However, neglecting the small increment of contrast with increasing the number of iterations, it can be seen that for all the reconstruction approaches, contrast enters a plateau region from about iteration 5 for qSMART and OSEM and 15 for MLEM.

As shown in Figure 4.e, the CNR calculated in images reconstructed by qSMART is larger than the one of MLEM and OSEM for iteration numbers less than eight regarding the relatively higher contrast values obtained by qSMART. However, with increasing the number of iterations, CNR of images reconstructed by qSMART rapidly decreases due to noise impact (Figure 4.c). OSEM reaches almost the same CNR as the highest CNR of qSMART after 19 iterations while CNR of MLEM slowly continues increasing.

NSE as a good figure of merit showing the similarity between the reconstructed image and the reference image was reported in Figure 4.f. Evidently, the lower NSE can be translated to better performance of the image reconstruction algorithm in generating an image more similar to the true image. As seen in Figure 4.f, in the first five iterations, qSMART resulted in smaller NSE values relative to MLEM and OSEM. However, at the sixth iteration, OSEM reaches the same NSE value as qSMART where qSMART starts generating worse NSE as the number of iterations increases. All the three methods could achieve almost the same NSE value but after different number of iterations; three iterations for qSMART, 35 iterations for MLEM, and 12 iterations for OSEM.

The reconstructed images of the micro-Derenzo phantom in Figure 5 reflect the performance of the reconstruction approaches for a rather complex phantom. As seen, although qSMART could reconstruct good images after three iterations, the reconstructed images get so noisy at higher number of iterations. On the other hand, MLEM and OSEM reconstructed acceptable images after respectively ten and three iterations while their best results occurred respectively after 20 and ten iterations. The highlight is that noise-induced degradation in the images of MLEM and OSEM with increasing the number of iterations is tolerable while this is not true in the case of qSMART.

Data discrepancy (quantified by MSE) against the number of iterations using the micro-Derenzo dataset was plotted in Figure 6.a for qSMART, MLEM, and OSEM. Regarding Equation 13, data discrepancy can show the convergence of the algorithm when no significant variation is seen in its value by increasing the number of iterations. Figure 6.a reflects what would be theoretically expected of the convergence speed of the three reconstruction algorithms. While qSMART and OSEM reached a plateau region, which can be translate to convergence, after about ten and 15 iterations, MLEM enters such a region at very higher number of iterations close to iteration 40. However, it should be remarked that although theoretically with increasing the number of iterations, MLEM and OSEM converge to a maximum likelihood solution (this can be translated to lower MSE value [Equation 13] for qSMART), but the convergence does not inevitably indicate that the resultant image is close to the true image (2, 21, 22). The convergence actually implies a small error between the forward projection of the obtained image and the measured projection. Also, it can be interpreted as no significant improvement in the reconstructed image is expected to happen after the convergence.

Figure 6b shows the bias versus noise curves for qSMART, MLEM, and OSEM obtained at different number of iterations. Considering the bias and noise as measures of respectively systematic error and random error in the reconstructed images, the best region in the graph is where both bias and noise have low values. As shown, MLEM reconstructed images of high bias at low number of iterations. But at higher number of iteration, it could reach a region with bias lower than 11% and STD% lower than 5%. Similar to MLEM, qSMART has large values of bias and noise at the first iteration. But, soon it could reach bias and noise values below respectively 11% and 6% after three iterations. The noise-bias curve, however, rapidly moves toward large values of noise with increasing the number of iterations. Overall, it is shown that OSEM and MLEM are quantitatively superior to the qSMART algorithm, achieving lower noise (bias) at a given bias (noise) value, as can be seen by drawing vertical (horizontal) lines in the noise vs. bias trade-off curves.

As shown in *Results* and discussed earlier in the current section, if qSMART is applied for image reconstruction of the PERSPECT, the reconstruction process should be halted at very low number of iterations (first five iterations) for the image quality is severely degraded at higher number of iterations. However, the results showed that qSMART cannot produce the best achievable images. Instead OSEM (and MLEM at high number of iterations) can reconstruct images of higher quality in comparison to qSMART with much degradation with increasing the number of iterations. Noting again is worth it that qSMART can also be considered as OSEM with subset size of one (16 subsets when having 16 views). Since OSEM was performed using four subsets, further studies can be performed analyzing the effect of different subset sizes/number of subsets.

## Conclusion

In this study, the performance of three developed image reconstruction approaches including qSMART, MLEM, and OSEM were assessed and compared for a novel desktop open-gantry SPECT system. Various phantoms were simulated and the acquired projection data were then reconstructed separately using each method by different number of iterations to evaluate the quality of the reconstructed images both visually and by a thorough set of quantitative figures-of-merit. The proposed approach in OSEM depicted best performance in terms of computational effort and achievable image quality and quantitation.
